# Carotid Endarterectomy in a Patient with Posterior Cerebral Artery Infarction: Influence of Fetal Type PCA on Atypical Clinical Course

**DOI:** 10.1155/2015/191202

**Published:** 2015-06-10

**Authors:** Mehmet Kolukısa, Azize Esra Gürsoy, Gülşen Kocaman, Hümeyra Dürüyen, Hüseyin Toprak, Talip Asil

**Affiliations:** ^1^Department of Neurology, Faculty of Medicine, Bezmialem Vakif University, Adnan Menderes Bulvari, Fatih, 34093 Istanbul, Turkey; ^2^Department of Radiology, Faculty of Medicine, Bezmialem Vakif University, Adnan Menderes Bulvari, Fatih, 34093 Istanbul, Turkey

## Abstract

Fetal type PCA may positively affect clinical outcome in patients with ischemic stroke such as protection of a reserve blood flow capacity as well as negative effect on clinical outcome such as certain atypical pathological manifestations. We presented a patient with a posterior cerebral artery territory infarction due to an unexpected etiology as stenosis of internal carotid artery (ICA) with existence of fetal type PCA.

## 1. Introduction

The immature structure of the fetal vertebrobasilar circulation is associated with the presence of anastomoses between the posterior and anterior systems. The most prominent of these anastomotic vessels, which exist only for a short duration of time (approximately 1 week) and undergo regression along with maturation of the vertebrobasilar system, is the posterior communicating artery (PComA). A higher adulthood diameter of PComA as compared to P1 segment of the posterior cerebral artery (PCA) is referred to as the fetal type PCA, which is reported in up to 30% of the adults affecting the blood supply in the circle of Willis [1]. In ischemic stroke patients, this condition may positively affect clinical outcome such as protection of a reserve blood flow capacity as well as negative effect on clinical outcome such as certain atypical pathological manifestations. In this report, we presented a patient with fetal type PCA and atypical clinical course. In the patient, we encountered an unexpected etiology due to fetal PCA.

## 2. Case

**Figure 1:**
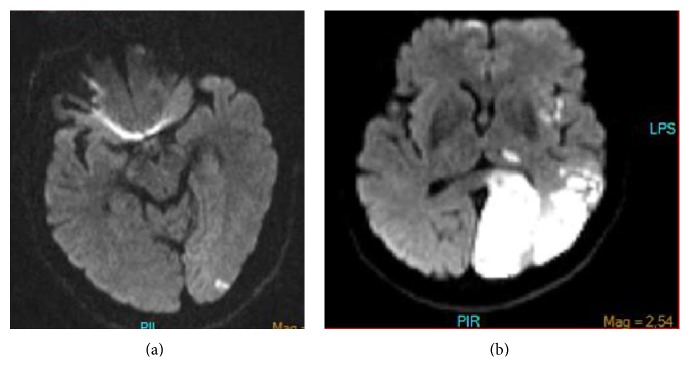
(a) Diffusion MRA showed small infarct on left posterior cerebral artery territory. (b) Diffusion MRA showed large infarct on left posterior cerebral artery territory and multiple infarcts areas on left middle cerebral artery territory.

**Figure 2:**
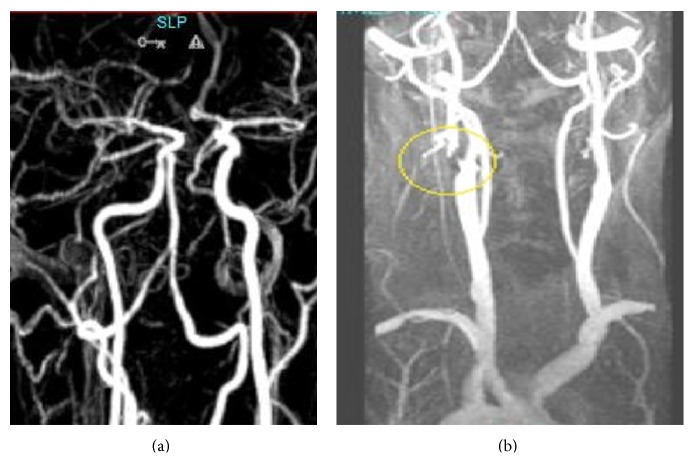
(a) Cranial MR angiography showed left fetal type PCA. (b) Cervical MR angiography showed critical stenosis on left proximal internal carotid artery.

A 63-year-old patient with a history of coronary stenting and regular use of antiaggregating agents presented with acute onset of dysarthria. Neurological examination was unremarkable except for dysarthria, and a diffusion weighted cranial magnetic resonance imaging (DW-MRI) showed a small occipital lesion only in left side. Although clopidogrel was added to his already existing antiplatelet treatment with ASA, his clinical condition deteriorated within several hours, and the patient had mild right hemiparesis and right hemianopia. A repeat DW-MRI showed the presence of simultaneous infarcts involving the territory supplied by the left PCA and inferior division of the left middle cerebral artery (MCA) (Figures 1(a)-1(b)). The cranial MR angiography demonstrated the presence of severe stenosis at the origin of the left internal carotid artery (ICA) in addition to fetal type PCA (Figures 2(a)-2(b)). Other etiological work-ups did not reveal further pathological conditions, and an early endarterectomy was performed. Following endarterectomy patient was stabilized and discharged with mild hemiparesis and hemianopia.

## 3. Discussion

A continuous transformation of the neural tube takes place during the development of embryo leading to continuous changes in the vasculature that assist in the adaptation to newly forming structures. The vasculature of the head and neck originates from the aortic arch. While during early fetal life the cerebral blood flow is mainly provided by the primitive ICA, the posterior cranium is supplied by the longitudinal neural artery. The latter, which is the origin of the future basilar artery, is connected to ICA via anastomoses [2]. These arteries, which are named after the cranial nerves they course along and which are referred to as “presegmental arteries” as a group, are represented by the otic (acoustic), hypoglossal, proatlantal, and trigeminal arteries and they connect the longitudinal arteries to the ICA. Their approximate life-span is one week and they disappear at eight weeks following the growth of PComA. Except for PComA, their frequency of occurrence after birth is approximately 0.1 to 1% as a coincidental finding, and the only anastomosis that persists following the birth is PComA. The other important embryonic issue is the portion of the intracranial vertebral retry from the PICA origin to the basilar artery. This part of the vertebral artery develops late and may be absent [3].

In adult type circulation, the diameter of P1, which is the segment of the PCA proximal to PComA, is greater than that of PComA and the blood supply of the occipital lobe is principally provided by the vertebrobasilar system. In cases where the diameter of PComA is greater than that of P1 or where P1 segment does not exist, the blood supply comes from the ICA via PComA. The reported frequency of this condition referred to as the “fetal type PCA” varies between 4% and 46% [4, 5]. The major factors responsible for this variance include the study populations involved (i.e., healthy adults versus patients with infarcts) and the imaging methods used (i.e., transcranial Doppler, MR angiography, or digital subtraction angiography). While it has been reported to exist in 56% of the autopsies performed in newborns, MRA screening yields a detection rate of 30% for fetal type PCA among healthy adults [1, 6]. A hypoplastic P1 segment is referred to as “partial fetal PCA,” while absence of P1 is defined as “complete fetal PCA” [7]. In such individuals who get the blood supply to their occipital lobe through ICA, this may offer certain advantages in atherosclerotic processes involving the posterior circulation, or the absence of P1 may prevent the occurrence of a posterior system infarction that results from embolism from cardiac or vascular pathologies [8]. However, an increased blood flow within the ipsilateral ICA has been shown to be associated with an increased progression rate of the atherothrombotic process [9, 10]. Thus, the effects of fetal type PCA is physiopathologically controversial. However, in the presence of fetal type PCA, particularly when there is narrowing or occlusion in the ipsilateral ICA or when the cerebral perfusion is impaired (e.g., due to hypotension), watershed infarctions may occur in the territory supplied by PCA. However, the presence of collaterals in Willis polygon may influence the development of those watershed infarcts. Again, the embolic infarctions in PCA territory may result from the atherothrombotic ipsilateral carotid artery [11]. Therefore, in patients presenting with simultaneous infarction of the posterior and anterior systems or in those individuals with posterior system infarction with a suspicion of embolism but without any obvious cardiac cause, the possibility of fetal type PCA, which is not an uncommon condition, should be kept in mind. In line with this, it is plausible to assume that, in our case presented above, embolic material moving distally from ICA through PComA early in the process might have subsequently resulted in the occurrence of infarctions in the left PCA territory. Hence, this patient with severe carotid stenosis had simultaneous posterior and anterior system infarctions while he was on effective antiplatelet treatment.

Fetal type PCA has also some clinical significance with respect to interventional procedures [12]. For instance, during endovascular procedures, the embolizing material may result in ischemia or infarction in the posterior circulation through these anastomoses, or during a carotid endarterectomy performed without adequate preoperative delineation of the anatomy of the circle of Willis and without ruling out the presence of permanent anastomoses, a clamp placed ipsilaterally may result in significantly reduced perfusion both in the cerebrum and in the brainstem.

## 4. Conclusion

Fetal type PCA is not an uncommon anatomic variation. It should be borne in mind particularly in patients with simultaneous infarctions in different vascular territories or in cases with atypical embolism where the nature of the infarction cannot be clearly defined. Examination of the presence of permanent fetal anastomoses by the preoperative use of noninvasive imaging modalities such as MR angiography or CT angiography may help prevent unexpected results.
